# Parliament and the Profession

**Published:** 1917-12-08

**Authors:** 


### Parliament and the Profession.

### East Leeds War Hospital.

THE Under-Secretary of State for War was asked by Mr. O'Grady if his attention had been drawn to a statement from British soldiers in the East Leeds War Hospital in reference to the difference of treatment of themselves as against that of an Austrian interned prisoner of war who, it was alleged, was fed with luxuries and treated with a consideration denied to wounded British occupants of the same ward. Mr. Macpherson said that the statements made respecting special treatment were incorrect, and explained the circumstances which gave rise to them.

### Experiments on Goats.

SIR G. GREENWOOD inquired of the Home Secretary as to the source of the funds to defray the cost of experiments on goats with poisonous gases and liquids, and as to whether anæsthetics were used. Sir George Cave explained that the experiments are carried out on behalf of the Ministry of Munitions, and that the cost no doubt fell upon public funds. In some cases anæsthetics were used, but in most of the experiments this was impossible without defeating the object of the experiments.

### Neurasthenic Soldiers.

IN answer to an inquiry by Mr. King, the Under-Secretary of State for War stated that the question of sending back to the Front soldiers who have suffered from neurasthenia has been discussed with the expert advisers, and the proposal is to classify neurasthenics on discharge from hospital in groups representing the periods during which they should not serve again in the firing-line.

### Hours of Work of V.A.D. Nurses.

MR. MACPHERSON informed Mr. T. Davies that nursing members of the Voluntary Aid Detachments in military hospitals work according to the time-table for regular military nurses (Queen Alexandra's Imperial Military Nursing Service). This time-table was circulated in the Official Report, and is as follows:

*Staff Nurses' Time-table.*—6.30 A.M., called; 7 A.M., breakfast; 7.30 A.M., wards; 9 A.M., light lunch, and dress; 9.30 A.M., wards; 12.30 P.M., lunch; 4 P.M. or 5 P.M., tea; 4.30 P.M. or 5.30 P.M., wards; 8 P.M., dinner; 10 P.M., bedrooms; 10.30 P.M., lights out.

*Times Off.*—Three hours every day from 9.30 A.M. to 12.30 P.M., or 2 P.M. to 5 P.M., or 5 P.M. to 8 P.M. Half a day every week from 2 P.M. to 10 P.M. A whole day every month from 6 P.M. the previous day to 10 P.M. the following day.

*Staff Nurses on Night Duty.*—Hours: From 8 P.M. to 7.30 A.M. A night off once a month. Two months' consecutive duty.

### The Factory Acts and V.A.D.s.

MR. T. DAVIES asked the Home Secretary whether his attention had been called to the conditions under which some of the Voluntary Aid Detachments are working in some public institutions, and whether he has any power to put the Factory Acts in operation where young women are not working under fair conditions. Sir George Cave stated that the Factory Acts do not apply to the employment of members of Voluntary Aid Detachments on nursing or domestic work in public institutions, and advised Mr. Davies to communicate with the Red Cross and St. John Ambulance Committee.

